# The validation of a new online cognitive assessment tool: The MyCognition Quotient

**DOI:** 10.1002/mpr.1775

**Published:** 2019-02-13

**Authors:** Anna C. Domen, Sjors C.F. van de Weijer, Monique W. Jaspers, Damiaan Denys, Dorien H. Nieman

**Affiliations:** ^1^ Department of Psychiatry Amsterdam University Medical Centers (location AMC) Amsterdam The Netherlands; ^2^ Department of Neurology Maastricht University Medical Center Maastricht The Netherlands; ^3^ Center for Human Factors Engineering of Health Information Technology, Department of Medical Informatics Amsterdam University Medical Centers (location AMC) Amsterdam The Netherlands

**Keywords:** assessment, cognitive functioning, neuropsychological tests, psychometrics

## Abstract

**Objectives:**

Cognitive impairment affects many psychiatric patients, influences daily functioning, and should be an important treatment focus. Assessment of cognitive status is crucial in cognitive remediation studies. However, current test batteries have limitations. A new, online tool, the MyCognition Quotient (MyCQ), was developed to assess cognition within 30 min. We present the psychometric properties and aim to determine the validity of the MyCQ by comparing it with the Cambridge Neuropsychological Automated Test Battery (CANTAB).

**Methods:**

Eighty‐seven patients diagnosed with obsessive compulsive disorder, schizophrenia/schizoaffective disorder, or major depressive disorder were included. Patients completed both the MyCQ and CANTAB.

**Results:**

Our hypothesized domains of psychomotor speed, attention, episodic memory, working memory, and executive functioning were confirmed by principal component analysis. The MyCQ total score correlated highly with the CANTAB total score. The MyCQ domains of psychomotor speed, attention, and episodic memory showed moderate to high correlations with corresponding CANTAB domains. Working memory and executive functioning had limited divergent validity.

**Conclusion:**

The MyCQ appears to be a promising instrument for assessing cognition online within a mixed psychiatric population. It is cost‐efficient, easily administered, and usable in different psychiatric populations, which makes it a good candidate for both clinical and community studies.

## 1 INTRODUCTION

Cognitive impairment is a common problem in psychiatric patients (Millan et al., 2012), seriously affecting functional outcome and quality of life in patients with a variety of psychiatric disorders (Brissos, Dias, & Kapczinski, 2008; Green, Kern, & Heaton, 2004; McCall & Dunn, 2003; Tolman & Kurtz, 2012). Treatment of cognitive deficits has received increasing attention, and cognitive remediation programs show promising results for patients with a psychotic disorder (Anaya et al., 2012; Grynszpan et al., 2011; Wykes, Huddy, Cellard, McGurk, & Czobor, 2011) as well as patients with other psychiatric diagnoses (Motter et al., 2016; Tchanturia, Lounes, & Holttum, 2014). To further develop and validate these cognitive remediation programs and for successful implementation in the (outpatient) clinic, assessment of the patient's cognitive status is essential. Unfortunately, although many good instruments are available for assessing cognitive functioning, their content, methods, and applications vary widely (Keefe et al., 2004). They require specialist supervision making them time‐consuming and expensive and are usually confined to a clinical setting. A new, web‐based tool, the MyCognition Quotient (MyCQ), has been developed to overcome these drawbacks. The aim of this paper is to present the MyCQ and report its psychometric properties.

To measure the full spectrum of cognitive functioning, traditionally, a broad range of separate tests are combined into a neurocognitive test battery. There is an extensive choice of tests available that have proven themselves valid, reliable, and sensitive over the last decades in measuring specific aspects of cognitive functioning. However, the combination of a sufficient number of these traditional tests into a complete test battery can lead to a lengthy assessment, taking up to several hours to complete. This can be burdensome for a patient and affect their motivation and performance, which might even lead to trial dropout. Furthermore, leaving researchers free to create their own test battery can result in a wide variety of test combinations. This negatively influences consistency and generalizability of the results, and thus hinders the standardized evaluation of new interventions aiming to improve cognition. This method also requires test supervisors to be proficient in many different tests. In the last decade, this issue has fortunately received more attention, and a number of guidelines and standard batteries for measuring cognition in psychiatric patients were developed. The Measurement and Treatment Research to Improve Cognition in Schizophrenia (MATRICS) Consensus Cognitive Battery for the assessment of cognitive functioning in schizophrenia (Nuechterlein et al., 2008) could now be considered the gold standard for cognitive assessment in schizophrenia, being recommended by the Food and Drug Administration for clinical trials of cognitive enhancement therapies in schizophrenia. The Brief Assessment of Cognition (BACS; Keefe et al., 2004) is another standardized comprehensive battery for schizophrenia, of which a new version is currently available for affective disorders (BAC‐A).

Nowadays, ease of access of test instruments is important for both research as well as clinical practice. Initially, cognitive assessment required the minimum involvement of pen and paper, but often also utilized various props such as colorful blocks (Wechsler, 2008) or plastic balls (Culbertson & Zillmer, 1998). There are currently a number of computerized neurocognitive test batteries available, such as the Cambridge Neuropsychological Test Automated Battery (CANTAB; Cambridge Cognition Ltd., 2008), CogStateBattery (https://http://www.cogstate.com), Cogtest (https://www.cogtest.com), and CNS vital signs (Gualtieri & Johnson, 2006). Furthermore, the BACS was recently transformed into a digitalized version in the form of an app that can be used on an iPad (Atkins et al., 2017). These computerized batteries have the advantages of having standardized and consistent administration and automated response recording and scoring, reducing errors in scoring. However, these computerized tests are still dependent on software and hardware that can be relatively expensive, are often tied to a specific location, and still require supervision by a trained expert. Web‐based and self‐administered assessment would increase accessibility and reduce costs, which could be advantageous for both research as well as clinical practice. In the last years, the mobile, self‐administered assessment of cognition has slowly started to make ground, but not many validation studies have yet been done. The THINC‐integrated tool (THINC‐it) cognitive screener app (McIntyre et al., 2017) has recently been validated for subjects with depression. In the current paper, we study an online and patient‐administered instrument for patients with varying psychiatric disorders, the MyCQ.

The MyCQ was developed to assess the broad cognitive status of patients in a relatively quick and easy manner. The instrument was developed with the primary care setting in mind. It is intended as a self‐administered tool, to cut down on clinical workload and open up cognitive assessment to larger numbers of patients. Originally, the MyCQ was intended to be used in unison with an online cognitive training application, where it tracks progress and helps determine which cognitive domains require most training. It furthermore could be used for research purposes as well as in care settings, fulfilling a screening function or to help keep track of cognitive status longitudinally. The MyCQ aims to assess five cognitive domains through 10 short subtests and can be completed online via PC or iPad. Furthermore, the MyCQ was developed to be used transdiagnostically, without a specific patient population in mind.

In the present study, we assessed the psychometric properties of the MyCQ in a transdiagnostsic sample. Internal consistency and structure of the instrument were evaluated. Convergent and divergent validity was determined by comparing the MyCQ with the CANTAB. Finally, we examined the ability of the cognitive domains to relate to age and premorbid IQ.

## 2 METHOD

### 2.1 Subjects

Subjects were recruited between May 2014 and February 2017 at the psychiatry department of the Amsterdam University Medical Centers (location AMC), The Netherlands, as part of a randomized controlled trial investigating the effectiveness of a new web‐based cognitive training game. This study was approved by the Medical Ethics Committee of the AMC and carried out in accordance with the latest version of the Declaration of Helsinki. Primarily, eligible patients coming for intake or treatment for their psychiatric disorder were invited to participate in the study. Subjects were also recruited by flyers and posters distributed throughout the AMC. To be included in the trial, subjects had to be aged between 16 and 55 and diagnosed with a main Diagnostic and Statistical Manual of Mental Disorders ‐ Fourth Edition (Text Revision) (DSM‐IV‐TR; American Psychiatric Association, 2000) Axis I disorder of schizophrenia/schizoaffective disorder, obsessive compulsive disorder or major depressive disorder. Exclusion criteria were a high risk of suicide, an unstable comorbid medical disorder, meeting the criteria for a substance use disorder within the last 3 months, a history of a clinically significant abnormality of the neurological system or seizure, and a premorbid IQ below 70. All subjects received a compensation of €40 and a travel cost reimbursement. Written informed consent was obtained from all subjects and from the legal guardians when the subject was younger than 18 years.

### 2.2 Materials

#### 2.2.1 The MycQ

The MyCQ was developed to assess cognitive functioning in a fast and efficient way. It is available online and was developed to be self‐administered in a variety of settings. The MyCQ consists of 10 subtests that are hypothesized to measure five primary cognitive domains: psychomotor speed, attention, episodic memory, working memory, and executive functioning. Although researchers do not fully agree on how cognitive functioning can be divided into different domains, the MATRICS initiative has identified seven separate cognitive domains (speed of processing, attention/vigilance, working memory, verbal learning and memory, visual learning and memory, reasoning and problem solving, and social cognition) that are often affected in schizophrenia and that are now leading in studies about cognitive function in schizophrenia (Nuechterlein et al., 2004). The MATRICS domains are similar to those of the MyCQ. Although the MyCQ considers episodic memory as one domain, the battery includes a verbal as well as a visual memory task. The MyCQ does not include a social cognition domain, because it focuses specifically on neurocognitive functioning. Social cognition is recognized as an important construct that is often impaired in schizophrenia (Green & Leitman, 2008) as well as in mood disorders (Ladegaard, Larsen, Videbech, & Lysaker, 2014) and is associated with functional impairment (Fett et al., 2011). However, social cognition and nonsocial neurocognitive functioning have been shown to be related but distinct phenomena (Fett et al., 2011; Hasson‐Ohayon, Goldzweig, Lavi‐Rotenberg, Luther, & Lysaker, 2018; van Hooren et al., 2008). With the five domains assessed by the MyCQ, the primary neurocognitive functions are covered. The MyCQ uses renowned paradigms that index the key areas of cognitive function. Each subtest is based on a reliable and well validated paper‐and‐pencil cognitive test. In the selection of the subtests, it was important that subtests were relatively brief and would be easy to use without expert administration. A detailed description of the subtests and their paper‐and‐pencil equivalents and the corresponding hypothesized domains are presented in Table 1 and Data S1. Written instructions with illustrative pictograms are included in the instrument and are provided prior to each subtest being taken. After the instruction, a short practice session is also provided. Subjects receive feedback on their performance after this practice in the form of the number of errors made and reaction speed and then proceed with the actual test. For every subtest, two outcome variables are recorded: mean latency and total number of errors, resulting in a total of 20 outcome variables.

**Table 1:** Individual MyCQ tests listed with test equivalents and corresponding cognitive domains

MyCQ subtest		Proposed domain	Validated test equivalent
1	Simple reaction time (SRT)	Psychomotor speed/attention	Donders type A
2	Choice reaction time (CRT)	Psychomotor speed/attention	Donders type B
3	Go no go reaction time (GNG)	Psychomotor speed/attention	Donders type C
4	Verbal memory recognition (VeMR)	Episodic memory	Rey auditory verbal learning test
5	Visual memory recognition (ViMR)	Episodic memory	Benton visual retention test
6	N‐back 1 (NB1)	Working memory	One back
7	N‐back 2 (NB2)	Working memory	Two back
8	Coding (COD)	Working memory	Digit symbol substitution test
9	Trail making test A (TMA)	Executive function	Trail making test part A
10	Trail making test B (TMB)	Executive function	Trail making test part B

*Note*. MyCQ: MyCognition Quotient.

#### 2.2.2 Cambridge Neuropsychological Test Automated Battery

To assess the convergent validity of the MyCQ, the CANTAB (Cambridge Cognition Ltd., 2008) was employed as second measurement of cognitive functioning. This computerized test battery has been extensively used in clinical practice as well as in scientific studies in a wide range of disorders and healthy controls. It is mentioned in over 2,000 scientific publications, and its ability to adequately discriminate between healthy adults and various (neuro) psychiatric populations has been confirmed (Egerhazi, Berecz, Bartok, & Degrell, 2007; Haring, Mottus, Koch, Trei, & Maron, 2015). It shows moderate correlations with traditional neuropsychological assessments (Smith, Need, Cirulli, Chiba‐Falek, & Attix, 2013). The CANTAB includes a variety of subtests that are delivered on a touchscreen computer and can be combined into different test batteries. We used the five CANTAB subtests choice reaction time (CRT), rapid visual information Processing (RVP), verbal recognition memory (VRM), spatial working memory (SWM), and intra–extra dimensional set shift (IED) to measure the five domains of psychomotor speed, attention, episodic memory, working memory, and executive functioning.

#### 2.2.3 Premorbid IQ

Premorbid intelligence was assessed by using the Dutch version of the National Adult Reading Test (NART; Bright, Jaldow, & Kopelman, 2002), which is a valid estimate of a person's premorbid level of intellectual ability. The NART is an untimed measure, consisting of 50 words with atypical phonemic pronunciation. Subjects are presented these words on a list and are asked to read each aloud.

### 2.3 Procedure

Subjects were assessed as part of a larger randomized controlled trial testing the effectiveness of a new cognitive remediation game, AquaSnap™. When approached for participation, subjects received written and oral information about the study goals and procedures. After signing informed consent and being negatively screened for the exclusion criteria, subjects were interviewed regarding their clinical, social, and cognitive status. Then, the cognitive assessment began with the NART. Thereafter, the subjects completed the CANTAB subtests followed by the MyCQ. The MyCQ was completed on a laptop with a mouse. A trained psychology student was present during the full assessment to introduce and supervise tasks and to make sure participants understood the instructions. Total testing time for each subject could take up to 4 hr. Because some subjects experienced difficulty sustaining their attention over such a period of time, sometimes two appointments were necessary to complete the measurement. The second appointment was always planned as soon as possible following the first appointment. Subjects could ask for a short break when needed during the testing session.

### 2.4 Statistical analyses

The underlying structure of the MyCQ was evaluated by examining intercorrelations and performing a principal component analysis (PCA). Internal consistency for the instrument and its subdomains were measured with Cronbach's coefficient.

Convergent and divergent validity was assessed by calculating Pearson correlations between the five composite domain scores of the MyCQ and CANTAB. Composite domain scores were computed by averaging the z‐scores for each domain. Some variables were inversed, so that a higher score meant more impaired cognition. The decision about which outcomes to combine into a composite score was based on factor structure for the MyCQ. For the CANTAB, we chose one subtest per cognitive domain and combined the key variables (CRT mean correct latency, RVPA and RVP correct rejections, VRM free recall (short term) and recognition (short and long term), IED total errors, and spatial working memory total errors) into a composite score.

We examined the sensitivity of the MyCQ to differentiate between in‐group differences by examining Pearson correlations between cognitive domain scores and sum scores and NART and age.

All statistical analyses were performed in SPSS (version 22) for Windows, and statistical significance was set at the 0.05 level.

## 3 RESULTS

A total of 87 subjects were included in this study (44 male). Age ranged from 16 to 56 years (*M* = 32.0, *SD* = 10.44). The mean total duration of the MyCQ was 30.8 min (range = 24.7–64.8, *SD* = 6.43 min). Table 2 presents the sample's demographic characteristics. Before further analyses, the data were inspected for outliers. Nine MyCQ and five CANTAB data points were identified as outliers for falling more than four standard deviations from the mean. These severely deviating data‐points most likely occurred due to measurement error (e.g., misplacement of the fingers on the keyboard or starting the subtest before instructions were clear) and were excluded from further analyses. Outliers were found in the MyCQ variables: simple reaction time total errors, N‐back 1 total errors, N‐back 2 mean latency, coding mean latency, trail making test A (TMA) mean latency, TMA total errors, and trail making test B (TMB) mean latency and in the CANTAB variables: RVPA, VRM short‐term recognition and long‐term recognition, IED, and CRT. Almost none of the subjects made errors on the MyCQ TMA and trail making test B (TMB), so both variables were excluded from further analyses. In Table 2, the means and standard deviations of the MyCQ variables are presented.

**Table 2:** Baseline demographic data and MyCQ outcomes of the sample, outliers removed

Characteristic	Mean (*SD*) or *n* (%)	Range
Age	32 (10.4)	
NART	99 (14.2)	
Gender		
Male	44 (50.6)	
Female	43 (49.4)	
Working?		
Yes	52 (59.8)	
No	34 (39.1)	
DSM‐IV‐TR diagnosis		
Psychotic disorder	36 (41.4)	
Obsessive–compulsive disorder	36 (41.4)	
Depressive disorder	15 (17.2)	
Level of education completed		
Higher tertiary	27 (31.0)	
Lower tertiary	20 (23.0)	
Secondary	34 (39.1)	
Primary	3 (3.4)	
None	2 (2.3)	
Unknown	1 (1.1)	
Marital status		
Unmarried	61 (70.1)	
Married or living together	21 (24.1)	
Divorced	4 (4.6)	
Unknown	1 (1.1)	
MyCQ subtest scores		
SRT Mean latency	375.9 (61.03)	258–564
SRT Total errors	1.0 (1.50)	0–7
CRT Mean latency	466.2 (93.79)	313–823
CRT Total errors	1.6 (2.85)	0–14
GNG Mean latency	510.4 (79.29)	371–793
GNG Total errors	1.4 (1.62)	0–6
VeMR Mean latency	931.0 (195.79)	582–1462
VeMR Total errors	11.4 (7.59)	0–37
ViMR Mean latency	848.9 (142.69)	544–1202
ViMR Total errors	8.8 (7.30)	0–33
NB1 Mean latency	796.0 (220.38)	461–1456
NB1 Total errors	2.7 (4.12)	0–27
NB2 Mean latency	1152.8 (338.59)	633–2384
NB2 Total errors	8.4 (7.15)	0–29
COD Mean latency	900.1 (217.17)	579–1865
COD Total errors	3.1 (2.97)	0–13
TMA Mean latency	888.4 (229.00)	530–1617
TMA Total errors	0.2 (0.66)	0–3
TMB Mean latency	1218.9 (399.63)	613–2646
TMB Total errors	0.6 (1.01)	0–4

*Note*. Mean latency in milliseconds. *SD*: standard deviation; NART: national adult reading test; SRT: simple reaction time; CRT: choice reaction time; GNG: go no go reaction time; VeMR: verbal memory recognition; ViMR: visual memory recognition; NB1: N‐back 1; NB2: N‐back 2; COD: coding; TMA: trail making test A; TMB: trail making test B; MyCQ: MyCognition Quotient.

### 3.1 Structure of the scale and internal consistency

To explore the underlying structure of the MyCQ, a PCA with oblique rotation was used on the 18 outcome measures. First, the correlation matrix was inspected to check appropriateness of the data. There were no variables that did not correlate with any other variable or that showed very high correlations with other variables. The determinant of the matrix was 6.972E‐5, meaning that there are no indications for multicollinearity in the data. Five components with eigenvalues greater than one were revealed. This model explained 68.0% of the total variance and largely confirmed our hypothesized domains of psychomotor speed, attention, episodic memory, working memory, and executive functioning. Four variables did not seem to be a perfect fit in the model. GNG total errors, N‐back 1 mean latency, and coding total errors did not show a high loading of >0.45 on any of the components. Furthermore, N‐back 2 Total errors showed similar loadings on two components (episodic memory and executive functioning). Repeating the PCA without these four variables resulted in the final five‐component model that had a good fit, including 14 variables. All included components had an eigenvalue above one; every variable presented a high loading on one component only; and the model explained 75.2% of the total variance and confirmed our hypothesized cognitive domains. Factor loadings and intercorrelations of the components of the final PCA are presented in Table 3.

**Table 3:** Factor structure of the MyCQ outcome measures

Final five‐component model					
	Psychomotor speed	Attention	Episodic memory	Working memory	Executive functioning
SRT mean latency	−0.917				
CRT mean latency	−0.664				
GNG mean latency	−0.899				
SRT total errors		0.840			
CRT total errors		0.721			
VeMR total errors			0.875		
ViMR total errors			0.904		
VeMR mean latency				−0.939	
ViMR mean latency				−0.883	
NB1 total errors					0.648
NB2 mean latency				−0.689	
COD mean latency					0.680
TMA mean latency					0.676
TMB mean latency					0.839
Variance explained	7.9	9.1	11.8	33.6	12.9
Correlations among components					
Psychomotor speed	‐				
Attention	0.198	‐			
Episodic memory	0.314^a^	0.191	‐		
Working memory	0.480^a^	0.091	0.123	‐	
Executive functioning	0.437^a^	0.220^b^	0.103	0.312^a^	‐
MyCQ sum score	0.774^a^	0.446^a^	0.538^a^	0.742^a^	0.619^a^

*Note*. SRT: simple reaction time; CRT: choice reaction time; GNG: go no go reaction time; VeMR: verbal memory recognition; ViMR: visual memory recognition; NB1: N‐back 1; NB2: N‐back 2; COD: coding; TMA: trail making test A; TMB: trail making test B; MyCQ: MyCognition Quotient. Pattern matrix and correlations between MyCQ domains. Principal Component Analyses with Oblimin rotation and Kaiser Normalization. Factor loadings <0.4 are hidden. Rotation converged in 12 iterations.

a Correlation is significant at the 0.01 level (2‐tailed).

b Correlation is significant at the 0.05 level (2‐tailed).

Internal consistency of the total MyCQ, including all 18 MyCQ variables, was good, with a Cronbach's α of 0.79. Internal consistency without the four items that were removed due to their low factor loadings was slightly smaller, with a Cronbach's α of 0.76. Cronbach's α was also computed for the specific cognitive domains as resulting from the PCA. These statistics were as follows: psychomotor speed, 0.86; attention, 0.37; episodic memory, 0.78; working memory, 0.73; and executive functioning, 0.69.

### 3.2 Convergent and divergent validity

**Figure 1:**
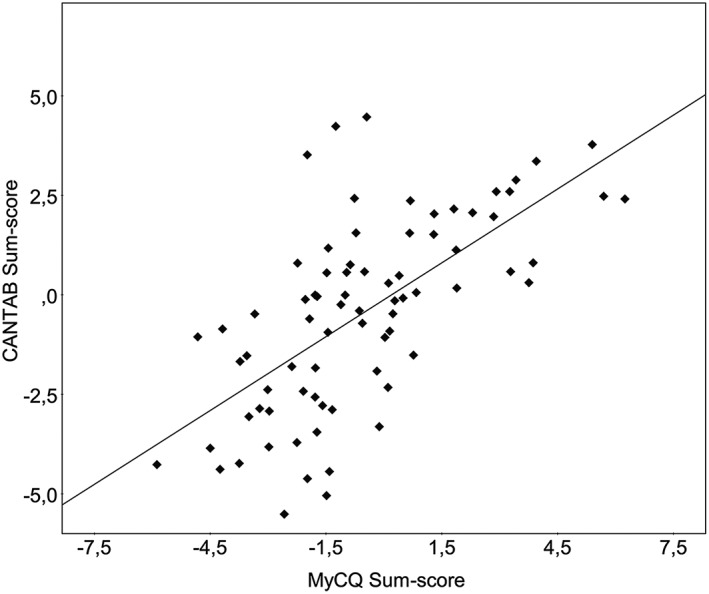
Scatterplot of the relation between MyCognition Quotient (MyCQ) and Cambridge Neuropsychological Automated Test Battery (CANTAB) sum scores (*r* = 0.664)

One subject was missing CANTAB data, this subject was excluded from further analyses. The MyCQ composites based on the PCA were compared with CANTAB composites using Pearson correlations. The results are presented in Table 4. Significant correlations were found between every matched domain, with the highest correlations between psychomotor speed (*p <* 0.001) and episodic memory (*p* < 0.001). All z‐scores were also summed to form one MyCQ or CANTAB sum score and were highly correlated (*N* = 77, *r* = 0.650, *p* < 0.001) as can be seen in Figure 1.

**Table 4:** Convergent and divergent validity: Pearson correlations between MyCQ and CANTAB domains

	CANTAB Domains					
		Psychomotor speed	Attention	Episodic memory	Working memory	Executive functioning
	Psychomotor speed	**0.604** a	0.311^a^	0.192	0.328^a^	0.243^b^
	Attention	−0.061	**0.224** b	0.065	0.182	0.172
**MyCQ Domains**	Episodic memory	0.194	0.374^a^	**0.374** a	0.353^a^	0.319^a^
	Working memory	0.371^a^	0.245^b^	0.123	**0.229** b	0.089
	Executive functioning	0.360^a^	0.432^a^	−0.066	0.442^a^	**0.278** b

*Note*. CANTAB: Cambridge Neuropsychological Automated Test Battery; MyCQ: MyCognition Quotient. Values printed in bold style correspond to convergent validity, values printed in regular style correspond to divergent validity.

a Correlation is significant at the 0.01 level (2‐tailed).

b Correlation is significant at the 0.05 level (2‐tailed).

For psychomotor speed and attention, divergent validity appeared to be reasonable, with lower correlations between nonmatching domains compared with the matching domains of CANTAB and MyCQ. Although the MyCQ domains of episodic memory, working memory, and executive functioning showed significant correlations with their corresponding CANTAB domains, these components showed comparable or stronger relationships with nonmatching CANTAB domains.

### 3.3 Associations between MyCQ scores and subject characteristics

Pearson correlations were calculated between MyCQ scores and age and NART with a significance level of 0.05. Higher age related to slower psychomotor speed (*r* = 0.224, *p* = 0.037) and worse performance on the working memory (*r* = 0.232, *p* = 0.033) and executive functioning (*r* = 0.393, *p* < 0.001). A higher NART score was associated with better MyCQ performance in psychomotor speed (*r* = −0.217, *p* = 0.044) and executive functioning (*r* = −0.254, *p* = 0.019).

In contrast, when comparing the CANTAB domain scores with these subject characteristics, only two significant correlations were found. The CANTAB sum score (*r* = 0.229, *p* = 0.038) and psychomotor speed domain (*r* = 0.223, *p* = 0.040) were significantly related to age, with higher age relating to worse performance. There were no associations between NART score and CANTAB domain or sum score.

## 4 DISCUSSION

The results of the current study provide initial evidence that the MyCQ is a valid and consistent instrument for a relatively quick assessment of cognitive functioning within a transdiagnostic psychiatric population. The instrument measures overall cognition within a relatively short time compared with more traditional instruments and can be self‐administered and accessed online, creating possibilities for increased accessibility and reduced costs.

The underlying structure of the MyCQ confirmed the hypothesized key cognitive domains reasonably well. The MyCQ subtests load into five separate components, which can be interpreted as the domains of psychomotor speed, attention, episodic memory, working memory, and executive functioning. Although no true consensus exists about which domains of cognition should be considered key domains, a few initiatives have attempted to bring more clarity. The DSM‐5 defines six key domains of cognitive functioning (perceptual‐motor function, language, learning and memory, social cognition, complex attention, and executive functioning; Sachdev et al., 2014), while the MATRICS initiative identified seven domains on which schizophrenia patients are impaired, including speed of processing, attention, working memory, verbal memory and visual memory, reasoning and problem solving, and social cognition (Nuechterlein et al., 2004). Although these domains do not fully conform to the domains of the MyCQ, there is much overlap, and the MyCQ includes tests that measure almost all of these domains. The five domains covered by the MyCQ should provide an adequate quick overall screening for neurocognitive functioning.

During the PCA, we dropped a few items that did not fit in well in the model. Fourteen of the 20 MyCQ outcome measures were eventually included in the final analyses. Most of these measures provide useful information, but the total number of errors of the TMT A and TMT B are an exception, for almost no errors were made on these subtests. In addition, errors on either of these subtests would probably result in a longer mean latency, indicating that this variable can safely be dropped from further analyses. Four more outcome measures were excluded due to either not loading highly on any of the retained factors or loading similarly high on more than one factor. Removing outcome measures that complicate the factor structure of an instrument is common practice. Simple structure, in which each item loads relatively strong on one factor and small on others, helps to achieve easy and meaningful interpretation (Thurstone, 1947). All subtests are still incorporated in the final MyCQ total score and subdomains, providing evidence that the five cognitive domains are covered by the 10 subtests of the MyCQ. More than 75% of the total variance observed was explained, a good result compared with other studies (Henson & Roberts, 2006). Nevertheless, our sample was quite small for this type of analysis, which could be of influence on the accuracy and generalizability of the model. Although opinions on minimum sample sizes for PCA differ, most rules of thumb ask for at least 10:1 ratio of N to variables (Osborne & Costello, 2004). Until the current results are replicated in a larger study population, caution in the interpretation of these domains is warranted.

Most MyCQ domains are moderately associated with each other. This finding is not surprising and has been noted by scientists since the beginning of the 20th century when Charles Spearmen first proposed the existence of a general intelligence factor, or *g* factor (Spearman, 1904). Furthermore, some of the subtests also load on different domains. For example, different outcome measures of the verbal and visual memory subtests are used for both the working memory and the episodic memory domain. The high association between working memory and psychomotor speed can be explained by both domains relying heavily on the mean latency of subtests.

Comparing the MyCQ with the CANTAB shows reasonable validity of the instrument. Sum scores of the MyCQ and CANTAB strongly relate to each other. All MyCQ domains show significant associations with corresponding CANTAB domains, with the strongest association between the psychomotor speed domains. This domain consists of the mean latency variables of the reaction time tasks (simple, choice, and go‐no‐go). MyCQ psychomotor speed relates to the CANTAB domains of attention, working memory, and executive functioning, but not as well as it does to the CANTAB psychomotor speed domain. Attention is covered by both the errors on the simple and choice reaction time task. Within the MyCQ, it appears to be especially specific, showing only a significant association with CANTAB attention, while the associations with any of the other CANTAB domains were very small. The total numbers of errors on the verbal and visual memory tests form the episodic memory domain. MyCQ episodic memory shows comparable associations of moderate strength with CANTAB episodic memory and attention. CANTAB working memory and executive functioning show somewhat smaller associations with MyCQ episodic memory. CANTAB psychomotor speed is not significantly related to MyCQ episodic memory. The working memory component includes four variables: the mean latency of the two memory tasks, the mean latency of the 2‐back task, and the errors on the coding task. This domain relates to the corresponding CANTAB domain, although this relationship is quite weak. Somewhat stronger associations exist with the domains of attention and psychomotor speed. The moderate correlation between working memory and CANTAB psychomotor speed might be explained by the fact that MyCQ working memory is largely based on mean latency outcome measures. Curiously, MYCQ working memory and CANTAB executive functioning are not related, although previous studies suggest that these are overlapping concepts (Chan, Shum, Toulopoulou, & Chen, 2008; McCabe, Roediger, McDaniel, Balota, & Hambrick, 2010), and the MyCQ also shows a moderate association between the two. Finally, the errors on the 1‐back task, the mean latency of the coding task, and both trail making tests' mean latencies are included to form the executive functioning domain. MyCQ executive functioning has a significant but small correlation with the corresponding CANTAB domain. Most other CANTAB domains are moderately associated with MyCQ executive functioning.

Although the MyCQ domains of psychomotor speed, attention, and episodic memory show decent divergent validity, this appears less the case for the domains of working memory and executive functioning. The concepts of executive functioning (and to a lesser extent working memory) are more complicated, broad and open to conjecture, than the concepts of attention, motor speed or episodic memory. As higher order domains, the structure of executive functioning and working memory are more difficult to capture, because they encompass a range of different components that can even be quite fractioned (Chan et al., 2008). In the current study, only one CANTAB subtest was used per cognitive domain. Although the CANTAB executive functioning domain in this study captured set‐shifting, the MyCQ domain consisted of four subtests, measuring different aspects of executive functioning. In a previous study, executive functions appeared to exist out of three latent structures, including one that was considered to be working memory (Lehto, Juujarvi, Kooistra, & Pulkkinen, 2003). Furthermore, a recent study showed that the CANTAB subtests themselves do not distinguish well between four cognitive domains found in traditional neurocognitive tests (Lenehan, Summers, Saunders, Summers, & Vickers, 2016). This highlights the overall difficulty of measuring human cognition. The poor divergent validity of the domains of working memory and executive functioning warrants some caution when interpreting the results of the MyCQ. Although using the MyCQ for assessing global cognitive functioning by using the overall score seems valid, for the confirmation of impairments in the separate domains—especially those of working memory and executive function—additional tests might be considered.

The MyCQ is somewhat sensitive to differences in age and premorbid IQ. Better MyCQ psychomotor speed and executive functioning scores are associated with a lower age and a higher premorbid IQ. Working memory also declines with age. In contrast, the CANTAB domains are not associated with premorbid IQ and only the sum score and psychomotor speed show age‐related decline. When using the Bonferroni correction for multiple comparisons, only the association between age and executive functioning retains its significance. However, the necessity to Bonferroni correct has been disputed. Especially with a relatively small sample size such as in our study, the probability of Type II errors greatly increases with this method, while the power to find small effects is reduced (Nakagawa, 2004). The effect‐size remains the same, however.

This study has a number of additional limitations. To further validate the MyCQ, it should be compared with a wider array of different (both computerized and paper‐and‐pencil) neurocognitive tests. Specifically, for further establishing concurrent validity, the MyCQ subtests should be compared with their paper‐and‐pencil equivalents. Second, no group of healthy controls and only patients with three different psychiatric disorders were included in this study. For an instrument that aims to be universal and used across a broad population, more subpopulations should be studied. However, the results do show that the MyCQ can be used to assess the five cognitive domains in the psychiatric conditions we studied. Because we did not include any healthy controls, we are unable to determine if patients are impaired. Administration did not involve counter‐balancing. Therefore, order effects were not controlled for and might have influenced the scores on the MyCQ. However, because the scores were primarily used for correlation analyses without evaluating the raw scores, this should not have much effect on the presented findings. Finally, this study only determined a proportion of the psychometric properties of the MyCQ. Studies aiming to assess the sensitivity to change or test–retest reliability are in progress. Further studies should also investigate if the MyCQ can predict real‐life cognitive functioning, clinical symptoms, or psychosocial functioning.

Despite these limitations, the advantages of assessing cognition with an online assessment tool such as the MyCQ are numerous. Computerized tests make consistent and precise administration and scoring possible, reducing measurement error and examiner bias. Computerized testing has been used before, but offering it through an online portal is a new development and could be the next step in neurocognitive assessment. One of the important advantages of self‐administered and online assessment, over and above the cost‐efficiency, is that it enables large scale assessment in a range of settings, both in the clinic and the community. This could be especially important for prevention studies and interventions. There is evidence that cognitive impairment can precede other clinical symptoms. This is especially true for psychotic disorders (Fuller et al., 2002), but might also be the case for mood disorders although results are still inconclusive (Allott, Fisher, Amminger, Goodall, & Hetrick, 2016). Neurocognitive functioning might well be a shared risk factor for overall mental health and global functioning. Although more studies are necessary to enlighten how cognition influences later functioning, early detection of impairment in neurocognitive functioning might be important and could be widely implemented with an online instrument. However, the usability of the MyCQ for psychiatric patients in settings outside the clinic should be further established. The MyCQ already has been used to assess large school populations of 600–800 students, confirming its usability in community studies for healthy populations (Ratto, Cliveden, & Sparrowhawk, 2017).

The MyCQ opens up the possibility of home assessment, which might especially be cost and time efficient and less burdensome for patients. The current paper did not investigate assessment at home, and it is still unclear how home assessment might affect the validity and reliability of the results. Uncontrolled confounders, such as distractions, assistance, or substance use, might interfere with reliable assessment. However, in some situations, home assessment might even provide an ecologically more valid estimation of a patient's cognitive functioning in their daily life. The effects on the reliability and validity of the MyCQ when assessing cognition at home should be further investigated. A number of subjects in this study also completed a follow‐up measurement of the MyCQ at home without problems, providing first support for the usability of the instrument for home assessment.

In summary, the MyCQ appears to be a promising instrument for assessing cognitive functioning online within a mixed psychiatric population. It compares reasonably well with the CANTAB, and its short duration and self‐administration capability potentially reduces health care costs. More studies with different populations, for example, Parkinson's disease (van de Weijer et al., 2016) and breast cancer patients with depression, and different neuropsychological assessment batteries are in progress to further validate the MyCQ and investigate its usability within other populations.

## Supporting information

Data S1. Supporting information
