# Supplementary material for: The validation of a new online cognitive assessment tool: The MyCognition Quotient
Source: Int J Methods Psychiatr Res. 2019 Feb 13;28(3):e1775. doi: 10.1002/mpr.1775 (PMC6850139; doi:10.1002/mpr.1775)
Supplement: Supplementary file 1 — Data S1. Supporting information [file MPR-28-e1775-s001.docx]

**SUPPLEMENTAL MATERIAL**

**Description of the MyCQ subtests**

The MyCQ is a short, online cognitive test-battery that can be completed on a desktop / laptop computer or an iPad. It consists of the following ten subtests:

*Simple Reaction Time (SRT)* – With this subtest, reaction time is assessed by repeatedly delivering a simple, single stimulus on which the subject has to respond. A red circle appears in the same location in the middle of the screen. The subject reacts by pressing the space bar (on computer) or tapping the screen (on iPad) as fast as possible when the stimulus occurs. The stimulus and response remain the same during the test, but there’s a variable and random interval between the response and the delivery of the next stimulus. The stimulus is delivered for 30 repetitions. The main trial phase is preceded by a practice phase of 5 repetitions. This test assesses general alertness and processing speed.

*Choice reaction Time (CRT)* – This subtest is similar to the Simple Reaction Time task, but with addition of a second stimulus and response. One of two stimuli is randomly presented at varying intervals and should be matched with the appropriate response. A red circle that appears on the left side of the screen must be matched by pressing or tapping the “z” button as fast as possible. A red triangle appearing on the right side of the screen must be matched by pressing or tapping the “m” button as fast as possible. Again, there are 30 repetitions in the main phase and 5 repetitions in the practice phase. This task measures general alertness, processing speed and divided attention.

*Go No Go Reaction Time (GNG)* – A subtest that is similar to the Choice Reaction Time task, in that there are two possible stimuli. One of the stimuli – a red circle in the middle of the screen – requires the subject to make a motor response by pressing the space bar or tapping the screen, whereas the other – a red triangle in the middle of the screen – requires the subject to withhold a response (no-go). The stimuli are delivered for 44 repetitions. The main trial phase is preceded by a practice phase of 8 repetitions. This task assesses general alertness, processing speed and inhibition.

*Verbal Memory Recognition (VeMR)* – In this subtest the subject is presented a series of 18 words to be remembered. After all the words have been presented, a new list of 72 words is subsequently shown. The subject has to respond to the words that are recognized from the first list by pressing or tapping the “m” button. The “z” button is pressed or tapped when the subject sees a new word that was not on the first list. Words on the new list may be semantically or phonologically similar to the words on the first list. The main trial phase is preceded by a practice phase presenting a series of 4 words to be recognized in a following series of 16 words. This task assesses verbal memory and learning.

*Visual Memory Recognition (ViMR)* – This subtest is similar to the Verbal Memory Recognition subtest, but uses visual stimuli instead of verbal stimuli. The subject is offered a series of 24 pictures of various items to be remembered. After all the pictures have been presented, a new list of 96 pictures is shown. The subject has to respond to the pictures that are recognized from the first list by pressing or tapping the “m” button. The “z” button is pressed or tapped when the subject sees a new picture that was not on the first list. Pictures on the new list may be similar to the pictures on the first list. The main trial phase is preceded by a practice phase presenting a series of 4 pictures to be recognized in a following series of 16 pictures. The task assesses visual memory and learning.

*N-Back 1 (NB1)* – On this subtest, the subject is presented a series of pictures. The subject is required to respond by pressing or tapping the “m” button when the stimulus matches the one from 1 step earlier in the series, and by pressing or tapping the “z” button when this is not the case. The main trial phase consists of a series of 50 pictures. The practice phase consists of 5 pictures. This task aims to capture working memory span and information updating.

*N-back 2 (NB2)* – This subtest is a more complex variant of the N-back 1 test described above. The subject is presented with a series of a total of 50 pictures and has to respond by pressing or tapping the “m” button the current stimulus matches the one from 2 steps earlier in the sequence. When the current picture does not match the picture that was presented 2 steps earlier, the subject must press or tap the “z” button. A practice phase consisting of 5 pictures precedes the main trial phase. This task aims to capture working memory span and information updating.

*Coding (COD)* – This subtest is a digit-symbol substitution test. The subject is shown four different symbols that match with the numbers 1 – 4 in the top of the screen. In the middle of the screen, the subject is presented one of the four symbols and is required to respond as quickly as possible with the number corresponding to that symbol. A new symbol is then presented. This task is pre-set to end after 120 seconds. A practice phase that lasts 15 seconds precedes the main trial phase. This task measures a broad range of cognitive domains, most of all working memory, processing speed and attention

*Trail Making Test A (TMA)* – This subtest is a visual and spatial search test that requires the subject to sequentially connect 25 encircled numbers in ascending order that are distributed on the screen. The subject can connect them by tapping them on iPad or using the mouse on the computer and is asked to work as fast as possible. The main trial phase is preceded by a practice phase with 8 numbers. This task measures attention and executive function.

*Trail Making Test B (TMB)* – This subtest is the B version of the Trail Making Test and measures the subject’s ability in task switching and control. The task is similar to the Trail Making Test A described above, except that the subject must now connect alternate numbers and letters in ascending and alphabetical order (e.g., 1, A, 2, B, 3, C, etc.). There are a total of 25 circles that need to be connected as fast as possible. The main trial phase is preceded by a practice phase with 8 stimuli to be connected. This test measures attention, executive functioning and set switching.
